# Wear Behavior of Austenitic Stainless Steel 308L Fabricated by Wire Arc Additive Manufacturing

**DOI:** 10.3390/ma19112207

**Published:** 2026-05-24

**Authors:** Saleh Alzughaibi, Youssef Alammari, Abdulrahman Alrumayh, Mohammed T. Alamoudi, Faisal J. Alzahrani, Hussam H. Noor, Khalid Alqosaibi

**Affiliations:** 1 Department of Mechanical Engineering, College of Engineering, Qassim University, Buraidah 51452, Saudi Arabia; s.alzughaibi@qu.edu.sa (S.A.); y.alammari@qu.edu.sa (Y.A.); a.alrumayh@qu.edu.sa (A.A.); 2 Advanced Materials Technologies Institute, Energy and Industry Sector, King Abdulaziz City for Science and Technology, Riyadh 11442, Saudi Arabia; 3 Department of Mechanical Engineering, College of Engineering-Rabigh, King Abdulaziz University, Jeddah 21589, Saudi Arabia; fjsalzahrani@kau.edu.sa; 4 Department of Mechanical Engineering, College of Engineering, Taibah University, Medina 42353, Saudi Arabia; 5 Department of Mechanical Engineering, College of Engineering, King Saud University, Riyadh 12372, Saudi Arabia; kalqosaibi@ksu.edu.sa

**Keywords:** Wire Arc Additive Manufacturing (WAAM), austenitic stainless steel, 308, wear, tribology, pin-on-disk, microstructure

## Abstract

**Highlights:**

- WAAM-fabricated SS308L showed optical microstructural features consistent with ferritic–austenitic solidification.
- Wear volume increased markedly with increasing normal load.
- Sliding speed had a comparatively minor effect on wear behavior.
- The specific wear rate remained on the order of 10^−4^ mm^3^/N·m.
- The COF of the SS308L disk/WC ball tribopair decreased with load and became speed-dependent at 15 N.
- The results indicate that load is the dominant factor controlling dry sliding wear of WAAM SS308L.

**Abstract:**

Wire Arc Additive Manufacturing (WAAM) has emerged as a cost-effective and high-deposition-rate technique for fabricating large-scale metallic components; however, the complex thermal history inherent to the process leads to heterogeneous microstructures that can significantly influence tribological performance. In this study, the dry sliding wear behavior of WAAM-fabricated austenitic stainless steel 308L (SS308L) was systematically investigated using a pin-on-disk configuration. The influence of applied normal load (1.5–15 N) and sliding speed (0.03–0.229 m/s) on wear volume, specific wear rate, coefficient of friction (COF), and tangential force was evaluated. Optical microstructural observations indicated features consistent with a ferritic–austenitic solidification structure, including regions resembling polygonal ferrite, Widmanstätten ferrite, and austenitic dendritic morphologies. Wear results showed that wear volume and cross-sectional area increased monotonically with increasing load, while the effect of sliding speed was comparatively less significant. The specific wear rate remained on the order of 10^−4^ mm^3^/N·m with minor variations across test conditions. The COF decreased with increasing load up to 10 N, followed by a speed-dependent response at higher loads. The findings demonstrate that load is the dominant factor governing wear behavior in WAAM SS308L, while microstructural heterogeneity may contribute to frictional stability and wear resistance. This study provides valuable insight into the structure–tribology relationship of WAAM stainless steels and supports the optimization of process parameters for wear-critical applications.

## 1. Introduction

The development of advanced manufacturing technologies and the optimization of existing processes have been longstanding research priorities in manufacturing engineering. Among these technologies, additive manufacturing (AM) has emerged as a transformative production paradigm since its introduction in the 1980s [1]. By enabling the layer-by-layer fabrication of complex geometries directly from computer-aided design (CAD) data, AM offers significant advantages over conventional manufacturing routes, including enhanced design freedom, reduced material waste, and improved material utilization efficiency [2]. To standardize terminology and processes, ISO/ASTM 52900 [3] classifies AM technologies into seven categories based on the energy source, material feedstock, and bonding mechanisms employed.

This study focuses on Direct Energy Deposition (DED), one of the seven AM categories, in which a concentrated energy source is used to melt and deposit metallic feedstock along a predefined toolpath to produce three-dimensional [4]. DED processes are commonly classified by the type of energy source (e.g., laser, arc, or electron beam) and the form of feedstock (e.g., powder or wire) [5,6]. Within this category, Wire Arc Additive Manufacturing (WAAM) employs an electric arc as the heat source and wire as the feedstock, offering a high deposition rate and material-efficient solution for medium- to large-scale metal components.

WAAM relies on arc-based welding techniques to melt and deposit metallic wire in a controlled layer-by-layer manner [7]. Compared with powder-based AM technologies, WAAM exhibits higher deposition efficiency, minimal material waste, and lower equipment and operating costs, making it particularly suitable for fabricating large structural parts and for repair and remanufacturing applications [8]. Recent developments in real-time process monitoring and adaptive control strategies have further improved the geometric accuracy and process stability of WAAM-fabricated components [9]. Nevertheless, WAAM processes are also associated with challenges such as residual stress accumulation, surface roughness, heat-induced distortion, and anisotropic microstructure development due to repeated thermal cycling [10].

A wide range of metallic alloys—including aluminum, titanium, nickel-based alloys, and stainless steels—are commonly used as WAAM feedstock materials [11]. Among these, austenitic stainless steel 308L is widely adopted due to its excellent weldability, corrosion resistance, and mechanical stability [12]. The alloy possesses a face-centered cubic (FCC) crystal structure that provides high ductility and toughness, making it suitable for components used in chemically aggressive and mechanically demanding environments [8,11]. WAAM-fabricated SS308L stainless steel components are increasingly considered for load-bearing and service-critical applications where wear resistance and structural integrity are essential. Typical applications include large structural members, refurbished shafts and flanges, pump and valve components, manifolds, and marine or energy-sector hardware subjected to sliding contact, abrasive environments, and cyclic loading. In such applications, surface wear, frictional damage, and microstructural stability under service conditions are decisive factors governing component reliability and service life [8,11].

However, the complex thermal history inherent to WAAM results in unique microstructural evolution in SS308L, including layered grain morphologies and local microstructural heterogeneity, which can influence mechanical and tribological performance [12,13]. Previous studies on WAAM-fabricated austenitic stainless steels have demonstrated that parameters such as heat input, interlayer thermal history, and build orientation strongly affect microstructure, hardness distribution, and deformation behavior [13,14]. These processing-related microstructural variations highlight the need for a better understanding of how WAAM processing conditions influence wear and fatigue resistance, particularly for components operating under severe tribological conditions [15].

Tribology, encompassing friction, wear, and lubrication, plays a critical role in assessing the durability and performance of additively manufactured components. Investigations on AM-produced steels and nickel-based superalloys have shown that processing conditions significantly affect wear mechanisms and frictional behavior [16,17]. For WAAM-fabricated stainless steels such as SS316L and SS347, heat input and cooling rate have been identified as key factors influencing tribological response under dry and elevated-temperature sliding conditions [18,19]. In the case of WAAM SS308L, previous studies indicate that thermal gradients can produce local microstructural variations along the build height, which may influence the wear response of the deposited material [12,13].

In sliding contact, the wear response is controlled not only by the bulk mechanical properties of the material, but also by the continuous evolution of the surface and near-surface region during testing. Local plastic deformation, debris formation, oxidation, material transfer, and the formation or breakdown of mechanically mixed layers can affect both the coefficient of friction and the wear rate. Recent tribology studies have shown that interfacial evolution plays an important role in controlling friction behavior. For example, Yi et al. reported that Ti_3_C_2_T_x_ MXene nanoflakes can promote very low friction through the in situ formation of a tribofilm at the sliding interface [20]. Similarly, Yang et al. showed that engineered DE@Mo_2_CT_x_ MXene bio-microcapsule nanofluids can improve tribological and machining performance by modifying the contact interface [21]. Although these studies were conducted under different material systems and lubrication conditions, they demonstrate the importance of contact-interface evolution in friction and wear behavior.

Despite growing interest in WAAM stainless steels, systematic studies on the dry sliding wear behavior of WAAM-fabricated SS308L remain limited, particularly regarding the effects of normal load and sliding speed. In addition, the heterogeneous solidification microstructure produced during WAAM may influence the friction stability and wear response of the deposited material. Therefore, the present study investigates the dry sliding wear and friction behavior of WAAM-produced SS308L stainless steel using a pin-on-disk configuration. The effects of applied load and sliding speed on wear volume, specific wear rate, coefficient of friction, and tangential force are examined and discussed in relation to the observed microstructural features.

## 2. Materials and Methods

### 2.1. WAAM Specimen Preparation

The experimental workpiece was manufactured from austenitic stainless steel 308L using a 0.8 mm ER308L stainless steel MIG wire supplied by MENAM Stainless Wire Public Co., Ltd. (Bang Phriang, Thailand). The wire was supplied according to JIS Z3321 YS308L/AWS A5.9 ER308L [22,23], and its nominal chemical composition range is summarized in Table 1.

**Figure 1:**
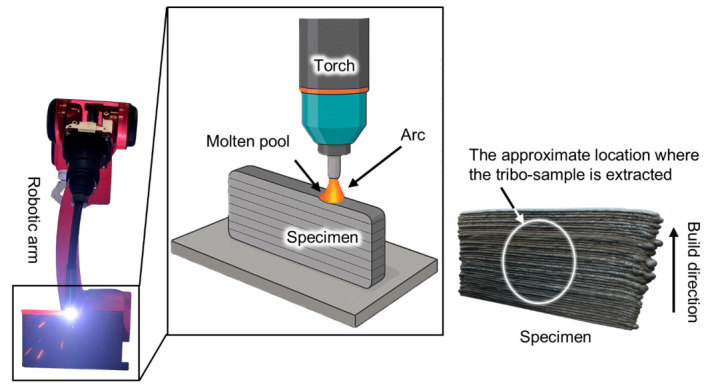
WAAM setup.

All specimens were produced using Wire Arc Additive Manufacturing (WAAM). The WAAM system comprised a Borunte six-axis robotic manipulator (Borunte Robot Co., Ltd., Dongguan, China) integrated with an Ehave CM350 arc-welding power source (Shenzhen Megmeet Electrical Co., Ltd., Shenzhen, China), as illustrated in Figure 1. The fabrication was carried out in the Additive Manufacturing Laboratory at Qassim University. A 0.8 mm-diameter 308L stainless steel wire was used as the feedstock, while a steel plate was used as the substrate. A vertically oriented rectangular wall, 220 mm long and 150 mm high, was deposited using the WAAM process. The printing parameters were set to a voltage of 20 V, a current of 140 A, and a travel speed of 3 mm/s. The nominal layer height was approximately 2 mm per deposited pass, and 75 layers were deposited to produce the 150 mm wall height. An interlayer dwell time was applied to allow the deposited wall to cool to approximately 100 °C before the next layer was deposited. High-purity argon (99%) was supplied as the shielding gas at a flow rate of 15 L/min. The resulting WAAM-fabricated plate is shown in Figure 1.

### 2.2. Metallographic Sample Preparation

Specimens of WAAM-fabricated stainless steel 308L were prepared for microstructural examination using standard metallographic techniques to characterize the as-built microstructure. Specimens were sectioned from the mid-thickness of the WAAM-deposited wall using a high-speed precision diamond saw (Buehler, Lake Bluff, IL, USA). The cut sections were mounted and ground using silicon carbide abrasive papers with progressively finer grit (SIA Abrasives Industries AG, Oberriet, Switzerland), reaching up to 2000 grit. Subsequently, the samples were polished using an alumina suspension with 0.5 μm particles to achieve a mirror-like surface finish. To reveal the grain boundaries and solidification phases, the polished surfaces were etched in a solution of hydrochloric acid and nitric acid (HCl:HNO_3_) at a 3:1 ratio for approximately 40–50 s. Microstructural analysis was conducted using an Olympus BX 41 M/LED optical microscope (Olympus Corporation, Tokyo, Japan), and the average grain size was calculated using the linear intercept method.

### 2.3. Wear Testing Setup

**Figure 2:**
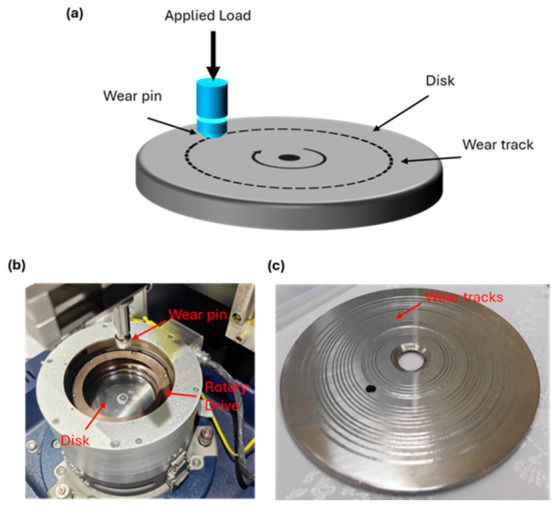
Overview of the pin-on-disk wear test procedure: (**a**) conceptual schematic of the test configuration, (**b**) Bruker UMT TriboLab equipment utilized in wear experiment, showing the wear pin positioned above the disk before engagement, and (**c**) surface wear tracks observed on the disk following test completion.

Pin-on-disk wear experiments were conducted using the Bruker UMT TriboLab universal mechanical testing system (Bruker Corporation, Billerica, MA, USA) in accordance with ASTM G99 [24] (G02 Committee, 2020). A fixed sliding distance of l_s = 100 m was maintained for all tests. To evaluate the influence of applied load on wear behavior, normal forces of F_N = 1.5 N, 2.5 N, 5 N, 10 N, and 15 N were applied. For every selected normal load, wear tests were conducted at three progressively increasing sliding velocities, namely 30 mm/s, 83 mm/s, and 229 mm/s, to examine the influence of sliding speed on wear performance systematically. This produced 15 force–speed conditions. The complete test matrix was repeated twice under identical conditions, giving two independent replicate wear tracks for each force–speed condition. Testing durations were adjusted accordingly to ensure a constant sliding distance across all conditions. The experimental configuration for the pin-on-disk wear tests is illustrated in Figure 2.

The wear experiments were conducted using WAAM-fabricated SS308L disks with a diameter of 68 mm, paired with 5 mm tungsten carbide balls as the pin material. Given the significant hardness difference between the WC ball and the SS308L disk, the disk was treated as the primary worn body in this study. Post-test optical inspection showed no obvious macroscopic geometric wear of the WC ball, although minor material transfer from SS308L to the WC ball was observed at higher loads. Therefore, the wear volume and specific wear rate reported in this work refer only to the SS308L disk. Accordingly, the reported coefficient of friction corresponds to the WAAM-fabricated SS308L disk/WC ball tribopair under dry sliding conditions at ambient laboratory conditions, rather than to SS308L as a standalone material property. Before testing, both components were carefully cleaned to avoid contamination; the balls were wiped with ethanol, while the disks were ultrasonically cleaned in ethanol for 5 min and subsequently dried with a hot air gun. To ensure uniform testing conditions, the disk surfaces were mirror-polished, achieving a surface roughness (Ra) of less than 0.8 µm. Surface roughness values were verified using a TR1900 tester (TES Electrical Electronic Corp., Taiwan, China), where three independent measurements were recorded and averaged for consistency. The mechanical and physical properties of the disk and pin are summarized in Table 2.

Wear experiments were conducted on a Bruker UMT TriboLab system (Bruker Corporation, Billerica, MA, USA). Two load cells were employed depending on the applied force: a DFM-1.0 cell (0.10–10 N range, 0.5 mN resolution) for tests at ≤5 N, and a DFM-20 cell (2–200 N range, 10 mN resolution) for tests at ≥10 N. Both load cells utilize a suspension mechanism to maintain stable force application. The system’s rotary drive allows for speed control across 0.1–5000 RPM, enabling precise adjustment of sliding velocity for all test conditions.

Pin-on-disk wear tests were carried out under controlled laboratory conditions (25 °C, ambient humidity). Test parameters were defined using an index system, where each index corresponds to a specific combination of applied load, sliding speed, and wear track radius. Sliding speeds ranged from 0.03 to 0.229 m/s, while the sliding distance was fixed at $l_{s}$ = 100 m for all tests. A summary of the test conditions is provided in Table 3.

**Figure 3:**
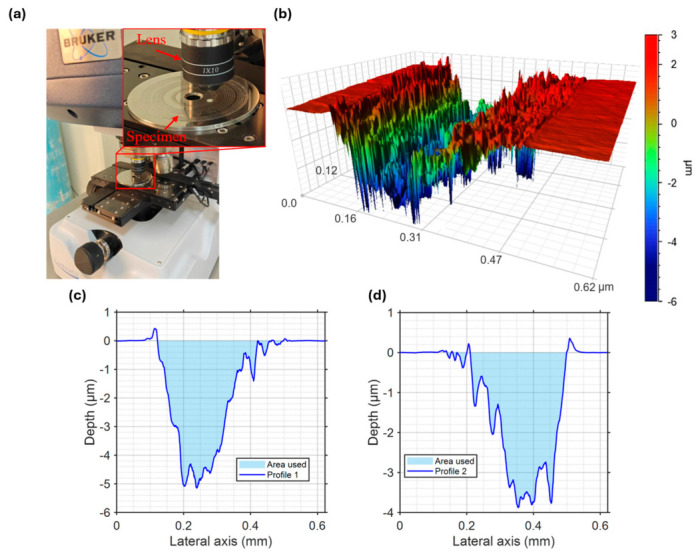
Profilometry setup and area-extraction workflow. (**a**) Bruker ContourGT 3D optical profilometer with specimen under the objective. (**b**) Representative 3D surface map of a representative wear track F2S83; color indicates depth (µm). (**c**,**d**) Two diametrically opposite cross-sections from the same track after shoulder-to-shoulder leveling; the shaded region is the valley integrated to obtain the cross-sectional area. The two areas are averaged to give the track mean.

Following completion of the wear tests, each disk was cleaned with ethanol to eliminate debris and surface residues. Wear scars were characterized using a Bruker ContourGT 3D optical profilometer (Bruker Nano Surfaces Division, Tucson, AZ, USA), as shown in Figure 3a.

For each track, a 3D surface map was acquired; a representative 3D surface map of wear track F2S83 is shown in Figure 3b. From each 3D surface, a single 1D profile was extracted by averaging the depth across the entire objective field of view (0.45 mm × 0.60 mm); two such width-averaged profiles were taken at diametrically opposite locations (see Figure 3c,d). Each profile was leveled by subtracting a linear shoulder-to-shoulder baseline, where values above zero were clipped to isolate the valley. The cross-sectional area A was obtained by trapezoidal integration of the positive depth. The two sections were averaged to yield the track mean, and their standard deviation was used as the uncertainty. The same procedure was applied to all tracks, and wear volume was calculated as $W_{V,disk}=A2\pi R$ using the measured track radius R. Finally, the specific wear rate of the SS308L disk was calculated following Archard’s model:

(1) $$k_{disk}=\frac{\left(W_{V,disk}\right)}{l_{s}\times F_{N}}$$

where $W_{V,disk}$ is the disk wear volume obtained from profilometry in mm^3^, $F_{N}$ is the applied normal load in N, and $l_{s}$ is the total sliding distance in m.

## 3. Results and Discussion

### 3.1. Microstructure Results and Discussions

**Figure 4:**
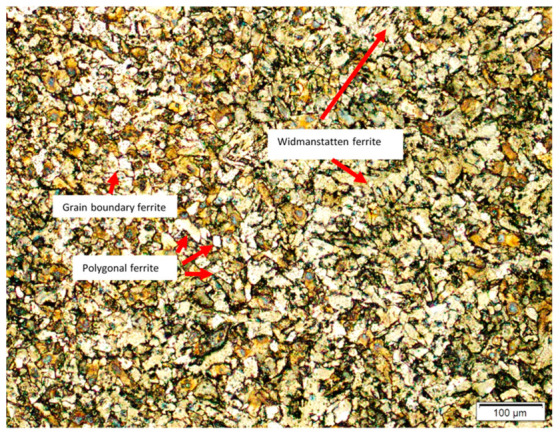
Optical micrograph of WAAM SS308L showing morphological features consistent with polygonal ferrite, Widmanstätten ferrite, and grain boundary ferrite structures.

Optical microstructural analysis of the WAAM-fabricated SS308L specimens indicated a complex, heterogeneous grain structure characteristic of the layer-by-layer deposition process. As shown in Figure 4, the observed morphology suggests the presence of ferritic features, including regions resembling polygonal ferrite, grain-boundary ferrite, and Widmanstätten-type ferrite.

**Figure 5:**
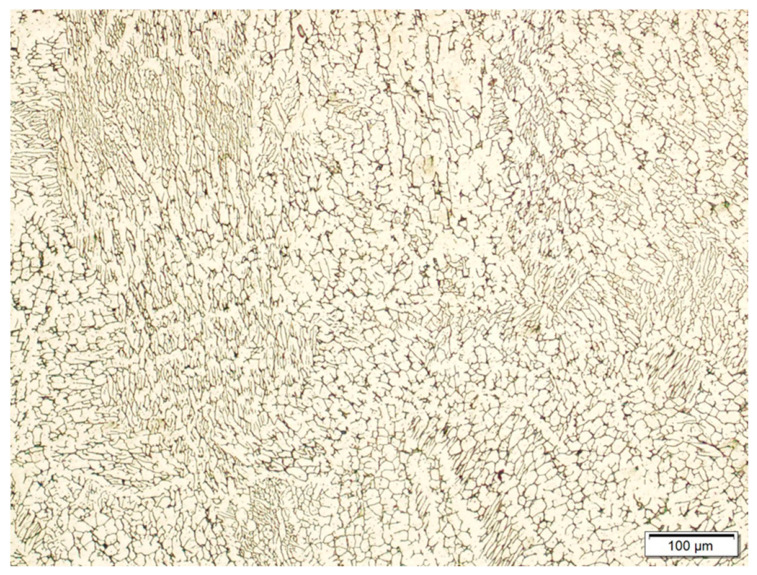
Optical micrograph showing dendritic regions with columnar, equiaxed, and cellular-dendritic morphologies, interpreted as austenitic regions based on optical morphology and literature comparison.

The average grain size was approximately 23.7 µm, with most grains exhibiting an equiaxed morphology. The presence of these specific ferritic structures suggests that the solidification process was governed by moderate cooling rates combined with the reheating effects of subsequent layers. In addition to the ferritic-like features, other regions showed dendritic morphologies that are consistent with austenitic regions reported for WAAM stainless steels. Figure 5 illustrates these regions, which display a mixture of columnar dendritic, equiaxed, and cellular-dendritic structures.

These morphological variations are a direct result of the thermal gradients inherent to the WAAM process, i.e., columnar dendrites typically form in areas with steep temperature gradients, while equiaxed grains nucleate in zones subjected to repeated thermal cycling. Cellular-dendritic structures likely emerged under intermediate thermal conditions. These coexisting morphological features suggest a ferritic–austenitic solidification structure in the printed 308L, although phase confirmation requires XRD analysis. It is worth noting the contrast between the 308L microstructure observed here and that of 316L stainless steel fabricated under similar WAAM conditions [26]. Previous studies on WAAM 316L indicate a predominantly austenitic structure containing retained δ-ferrite with skeletal and lathy morphologies. In 316L, the δ-ferrite solidifies first and partially transforms to austenite, creating a finer duplex distribution. This difference is attributed to composition: the higher nickel and molybdenum contents in 316L stabilizes the austenite phase, whereas the chemistry of 308L favors ferrite retention. Consequently, the WAAM 308L produced in this study exhibits a stronger tendency toward ferrite stabilization, manifested as polygonal and Widmanstätten forms, which may enhance resistance to solidification cracking but could induce anisotropy that influences wear performance.

It should be noted that phase identification in the present study is based on optical morphology and comparison with reported WAAM SS308L microstructures; therefore, XRD analysis is recommended in future work to confirm the ferrite/austenite phase constitution more rigorously.

### 3.2. Wear Test Results

**Figure 6:**
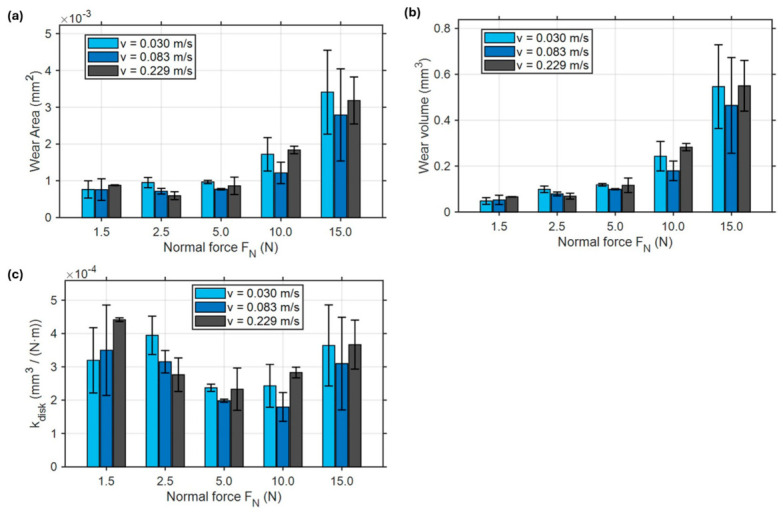
Quantitative wear metrics as a function of normal force *F*_N_ and sliding speed *v*. (**a**) Mean wear area from profile integration. (**b**) Mean wear volume computed as $W_{V,disk}=A2\pi R$. (**c**) Mean specific wear rate computed as $k_{disk}=\frac{\left(W_{V,disk}\right)}{l_{s}\times F_{N}}$. Bars represent the mean of two independent replicate wear tracks for each force–speed condition, and error bars represent the standard deviation between the two trials.

Two independent dry pin-on-disk replicate test series were performed at five nominal normal forces (1.5, 2.5, 5, 10, and 15 N) and three sliding speeds (0.030, 0.083, and 0.229 m/s), with a fixed sliding distance of 100 m. Each replicate series included the full experimental matrix, giving two independent wear tracks for each force–speed condition. For every track, two width-averaged cross-sections were extracted from the 3D profilometry, leveled, and integrated to obtain the cross-sectional wear area. The disk wear volume and the disk-specific wear rate were then computed. Figure 6 summarizes the quantitative wear metrics as functions of normal force F_N_ and sliding speed v. As illustrated in Figure 6a, the cross-sectional wear area increases monotonically with load for all speeds, rising from sub-10^−3^ mm^2^ at 1.5 N to a few ×10^−3^ mm^2^ at 15 N. At a given F_N_, differences among the three sliding speeds are small compared with the load effect; the intermediate speed v = 0.083 m/s tends to yield slightly larger areas at the highest load. Error bars grow with F_N_, indicating greater scar heterogeneity at higher loads. Similar to area, wear volume (Figure 6b) increases noticeably with F_N_ while the speed effect remains secondary. As expected, the error bars are larger at higher loads because they inherit the scatter from the area measurements. The specific wear rate (Figure 6c) is on the order of 10^−4^ mm^3^/N·m and varies slightly with F_N_ and v.

**Figure 7:**
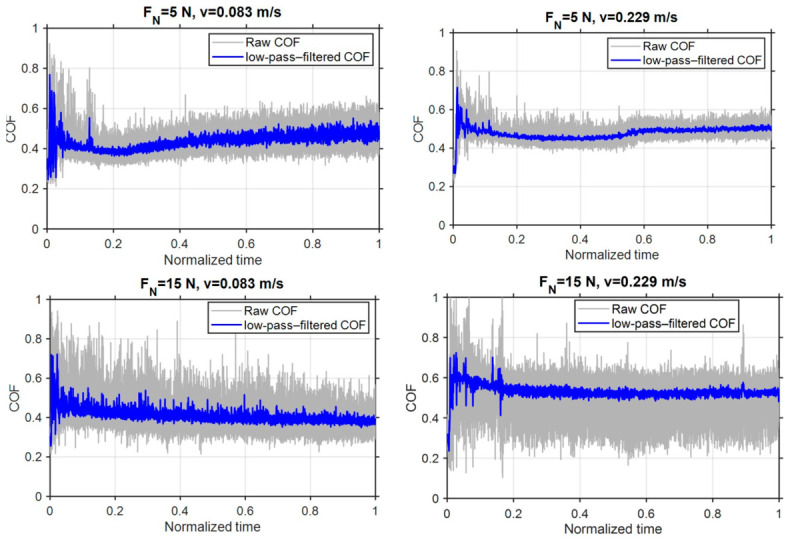
Coefficient of friction (COF) versus normalized time for representative conditions. Time is normalized from test start to end. Gray curves show raw COF; blue curves show COF after 0.1-Hz low-pass filtering.

To illustrate how the COF and F_R_ values are obtained, Figure 7 shows a representative COF record for representative cases. For each run, the COF was denoised with a 0.1-Hz low-pass filter, and the steady-state mean was computed over the last 70% of the normalized test time (i.e., *t*_norm_ ≥ 0.30). The same procedure was applied to the tangential force *F*_*R*_. This procedure yields the mean COF and mean *F*_*R*_ trends as functions of normal load and sliding speed, with variability reflecting trial-to-trial repeatability rather than within-trace noise.

**Figure 8:**
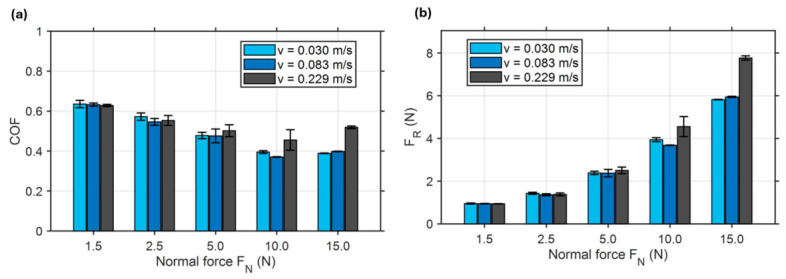
Friction metrics vs. normal force *F*_*N*_ at three sliding speeds. (**a**) Steady-state coefficient of friction (COF) versus normal force F_N_. Bars show the mean of the 0.1-Hz low-pass-filtered COF over the steady region (t_norm_ ≥ 0.3). (**b**) Steady-state tangential force F_R_ versus F_N_ for the same speeds (means over the same steady window). Error bars represent the standard deviation between the two independent replicate wear tracks for each force–speed condition.

Across the tested conditions, two trends emerge clearly. The coefficient of friction (COF) of the WAAM SS308L disk/WC ball tribopair drops as the normal force increases from 1.5 to 5 N, remains comparatively low at 10 N, and shows a distinct speed dependence at 15 N, with the highest speed yielding the largest COF, as shown in Figure 8a. The increase in COF at 15 N and 0.229 m/s may be attributed to the more severe contact conditions resulting from the simultaneous increases in load and sliding speed. Under this condition, the contact interface is expected to experience higher frictional heating and more intense debris interaction. This may reduce the stability of the mechanically affected surface layer and increase resistance to sliding, leading to a higher COF. Therefore, although the overall wear volume is primarily governed by normal load, the friction response at the highest load can still be affected by sliding speed through changes at the contact interface.

The tangential force *F*_*R*_ scales almost linearly with *F*_N_ for all speeds (see Figure 8b), indicating a predominantly Coulomb-like response. Any speed influence is modest and becomes noticeable only at higher loads.

## 4. Conclusions

This study investigated the microstructure and dry sliding wear behavior of WAAM-fabricated SS308L stainless steel under varying normal loads and sliding speeds. Based on the experimental results, the following conclusions can be drawn:

1. Optical microstructural observations indicated heterogeneous features consistent with ferritic–austenitic solidification, including regions resembling polygonal ferrite, grain-boundary ferrite, Widmanstätten-type ferrite, and dendritic austenitic morphologies. XRD analysis is recommended in future work to confirm the phase constitution more rigorously.
2. Wear behavior was strongly dependent on the applied normal load. Both wear area and wear volume increased monotonically with increasing load, indicating that load is the primary controlling parameter in the tribological response.
3. The influence of sliding speed on wear performance was secondary compared with load, with only minor variations observed in wear metrics across the tested velocity range.
4. The specific wear rate remained relatively stable (~10^−4^ mm^3^/N·m), suggesting consistent wear resistance of WAAM SS308L across different operating conditions.
5. The coefficient of friction decreased with increasing load up to 10 N, indicating improved interfacial stability, while at higher loads (15 N), a more pronounced dependence on sliding speed was observed.
6. The near-linear relationship between tangential force and normal load confirms a predominantly Coulomb-type friction behavior.

Overall, WAAM-fabricated SS308L exhibits stable, predictable tribological performance under dry sliding conditions, making it a promising candidate for structural and wear-resistant applications. Future work should include a separate and systematic investigation of build orientation, post-processing treatments, and high-temperature sliding conditions to further clarify their influence on the wear response of WAAM-fabricated SS308L.

## Figures and Tables

**Table 1:** Nominal chemical composition range of the ER308L wire feedstock used in this study.

Element	C	Cr	Ni	Mn	Si	Mo	P	S	Fe
wt.%	≤0.03	19.5–22.0	9.0–11.0	1.0–2.5	0.30–0.65	—	≤0.03	≤0.03	Balance

**Table 2:** Properties of the test specimen in the pin-on-disk test.

Test Specimen/ Material	Ball/Tungsten Carbide	Disk/WAAM-Fabricated SS308L
**Chemical composition mass fraction in %**	WC: 94 Co: 6	
**Hardness**	(90 to 91.5) HRA (1400 to 1550) HV	(85 to 88) HRB
**Mean surface roughness Rz [µm]**	--	
**Mean surface roughness Ra [µm]**	0.02 *	

* According to DIN 5401:2002 [25] (G10).

**Table 3:** Pin-on-disk test parameters.

Index	Normal Force, F_N_	Sliding Speed, v	Wear Track Radius, R
	[N]	[mm/s]	[mm]
F1S30	1.5	30	10
F1S83	1.5	83	11
F1S229	1.5	229	12
F2S30	2.5	30	16.5
F2S83	2.5	83	17.5
F2S229	2.5	229	18.5
F5S30	5	30	19.5
F5S83	5	83	20.5
F5S229	5	229	21.5
F10S30	10	30	22.5
F10S83	10	83	23.5
F10S229	10	229	24.5
F15S30	15	30	25.5
F15S83	15	83	26.5
F15S229	15	229	27

## Data Availability

The original contributions presented in this study are included in the article. Further inquiries can be directed to the corresponding author.
